# NLP-Based Digital Forensic Analysis for Online Social Network Based on System Security

**DOI:** 10.3390/ijerph19127027

**Published:** 2022-06-08

**Authors:** Zeinab Shahbazi, Yung-Cheol Byun

**Affiliations:** Department of Computer Engineering, Major of Electronic Engineering, Institute of Information Science & Technology, Jeju National University, Jeju 63243, Korea; zeinab.sh@jejunu.ac.kr

**Keywords:** digital forensics, natural language processing, blockchain, machine learning

## Abstract

Social media evidence is the new topic in digital forensics. If social media information is correctly explored, there will be significant support for investigating various offenses. Exploring social media information to give the government potential proof of a crime is not an easy task. Digital forensic investigation is based on natural language processing (NLP) techniques and the blockchain framework proposed in this process. The main reason for using NLP in this process is for data collection analysis, representations of every phase, vectorization phase, feature selection, and classifier evaluation. Applying a blockchain technique in this system secures the data information to avoid hacking and any network attack. The system’s potential is demonstrated by using a real-world dataset.

## 1. Introduction

Social media is generally used to communicate on the internet through various channels, to collaborate with different users, and to share information. The shared content supports researchers in investigating the potential of the criminal process. Social media does not have any limitations for content sharing related to victims, suspects, and witnesses [1,2]. The websites and applications are used to facilitate the sharing of content between connected networks. One of the social structures is the online social network (OSN), which which includes platforms such as Twitter or Facebook [3,4,5,6]. Forensic data extraction from social media platforms has become a considerable research problem [7,8,9]. Conventional digital forensics collects most of the information, which is a huge art of proof. Nevertheless, the extraction process is not practical on the OSN regarding the nature of the highly distributed network, shared content, and data size. Data collection from the individual subjects without any acceptable reason is almost unmanageable, and because of privacy laws, limited access is permitted [10,11,12]. Forensic data collection connects to the system operator for the formatting issue and data authenticity. The available digital forensics (DF) methods entail many challenges in cyber-physical systems. This includes the difficulties of data access, data originating from various locations, the traceability and transparency of evidence, and huge-volume data analysis. During the past few years, a large number of researchers have focused on forensic analysis based on cloud computing [13,14,15], evidence modeling [16,17,18], and assisting the community of law enforcement. Blockchain technology is a distributed ledger system that collects and saves the proper records in the decentralized format of a peer-to-peer network. The stored data are based on a timestamp block, and directly link with the chain based on proof of trust [19,20,21]. The advantages of applying blockchain in the DF system are to provide the digital evidence the accessibility of self-verification for ensuring the hash function and evidence chain verification. This process guarantees the system transparency, security, and immutability in case of examination. In this paper, we propose a digital forensic platform using the integrated method of NLP and blockchain, feature selection using machine learning techniques, network vectorization, and system security analysis. Moreover, the presented system focuses on the relationship between content communication and individuals. The system uses the supervised NLP for topic extraction and applies the feature selection for topic ranking to find the highly weighted topics. Regarding the ranked topics, the classifiers can train with the famous algorithms and generators, and the output will be effective classifiers that can modify various metrics for further investigation. The main contributions of this paper are summarized as below:

- This research applies natural language processing techniques for the detailed data analysis approach;
- One of the important aspects of this research is the multi data source input, which makes this process more competitive with other research results;
- The main focus of this research is a system security method which stores the OSN information in blockchain framework.

The remainder of this process is summarized in Section 2, which reviews the recent related literature and the current state of the art. Section 3 presents the details of the proposed digital forensics approach and a performance evaluation. Section 4 presents details for the results, the implementation of the proposed digital forensics analysis, and finally, the conclusion.

## 2. Related Work

In this section, the state of the art in DF is presented in detail. The main focus is on two parts. One is digital forensics challenges in blockchain, and the other is the forensic attainment of social media content.

### 2.1. Digital Forensics Challenges in Blockchain

In DF, hash functions are applied to maintain digital integrity and generate the digital digest to avoid changes of digital assets [22,23]. Nevertheless, in the applications related to DF, the main focus is on disk drive integrity and the validation of data. The biggest concern is the hash validation and verification for special files such as images. The DF approach depends on the investigators’ experience [24,25]. Some of the challenges related to the existing DF are presented as trustworthiness, integrity, provenance improvement, scalability, and availability [26,27]. In terms of trustworthiness, the system is supposed to check the trust if insider threats to the blockchain environment improve the trust of evidence [28]. Regarding integrity, the system checks the events examinations and items in the digital investigation. A traditional investigation provides forensic activities and supports the data, tools, etc. The improvement of provenance fetching at the top of hash functionality gives the information of hash validation to examine the system’s behavior with creating the hash tree. The scalability in the hash tree is able to support nodes of the system, and it is capable of the hash digest in the deep level [29,30,31]. Every blockchain node contains the whole hash information, guaranteeing accuratcy. This aspect saves the digital data for investigating forensic events.

### 2.2. Forensic Attainment of Social Media Content

The DF attainment involves steps of proofing the criminal cases regarding location, security, data, etc. [32]. The data provided from social media is more understandable and easy to access for users [33,34,35]. To use this type of data, it is required to follow the legal and formal process [36]. This process is performed by a highly skilled person with sufficient knowledge of technical and legal matters [37,38,39]. The artifacts of DF identify the critical sources for social media evidence [40,41]. Thus, lots of research materials focus on the attainment of forensic evidence; the extraction of forensic information from social media concentrates on the identification of specific devices and detects the traces found by the device from the web browsers or media applications [42,43,44,45]. To collect the forensic data, the requirements are defined as relevant data collection from multiple websites, metadata collection from social media information, and certifying the data integration in the forensic collection [46,47]. DF footage is mostly used for the comparative analysis of images and objects to find the relative subjects to provide the opinion findings [48,49,50,51]. Table 1 shows the existing state of the art in forensic data analysis. The main focus is on the research approach, limitations, and advantages analyzed in DF. The selected works show various DF analyses based on machine learning, video clustering, chat data encryption, instant message analysis, etc.

## 3. Proposed NLP-Based Digital Forensic Analysis for Online Social Network

**Figure 1:**
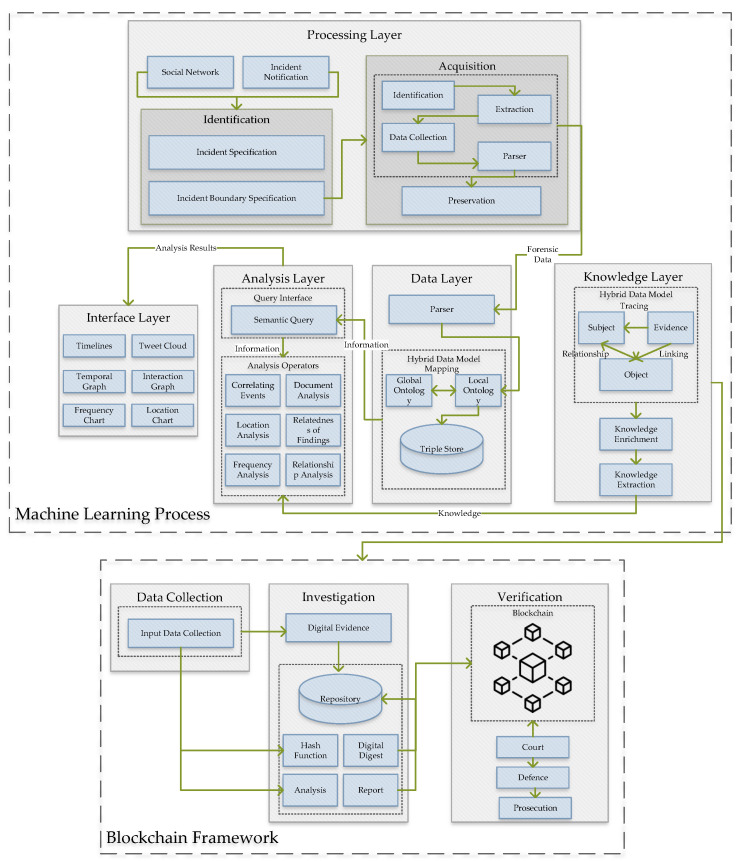
Overview of the proposed digital forensic analysis.

This section briefly presents the integration of NLP techniques with blockchain. Figure 1 shows the overview of the proposed digital forensic analysis in terms of the NLP and blockchain approach. The main goal of this system is to improve the security of the DF analysis regarding the information shared on social media.

NLP techniques are applied to analyze the collected dataset from every aspect to provide meaningful information to the proposed system. This process has five main layers: a processing layer, an interface layer, an analysis layer, a data layer, and a knowledge layer. The responsibility of the processing layer is to identify and acquire the system input. Inputs are from social networks and incident notifications for which identification requires the incident specification and incident boundary specification. The identification, extraction, data collection, the parser, and preservation are required before moving forward to acquisition. Completing this process, the forensic data is the input of the data layer. In this layer, the hybrid data mapping is used for global and local ontology and storing the data. The next layer is the analysis layer for which the interface query is an NLP semantic interface, and the analysis operators are correlation events and document analysis, location analysis, the relatedness of findings, frequency analysis, and relationship analysis. The analysis report moves to the interface layer, which contains the timelines, tweet cloud, temporal graph, interaction graph, frequency chart, and location chart. Next is the knowledge layer, where the relationship between the extracted dataset and its linking is processed. After completing the NLP steps and data processing, the analyzed dataset is ready to save into the blockchain framework. The main reason for using the blockchain framework is to secure the collected dataset with limited access to avoid hacking or attack. The blockchain framework contains the data collection, investigation, and verification processes, which provides the verified data to a court for defense and prosecution.

### 3.1. NLP-Based Digital Forensic Analysis

**Figure 2:**
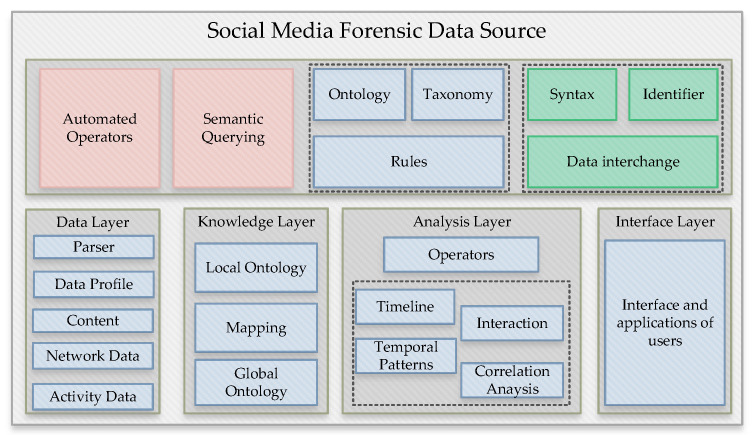
Multi-layered implementation process.

The presented knowledge model is an event-based system that prepares the social media analysis based on electronic forensics. The ontology technique is applied for representing the related knowledge of OSNs. The detailed explanations show the automated method process and provide formal information through ontologies for the true validation and automated techniques. The investigation of forensic models is from the collection of semi-automated processes. This model contribution provides the boundary identification of data collection from the social media distribution network. The model gives the limitations of forensic data in terms of appropriate parameters and automated collection. Figure 2 describes the details of every layer for the forensic data-analysis process: the automated operators, semantic querying, the rules of the ontology and taxonomy processes, and the identification of the data interchange regarding the defined layers processed for analysis of forensic data system. The data layer contains the parser, the data profile, the content, the data from the network, and activity data. The knowledge layer contains the local/global ontology process and mapping details. The analysis layer gives the information and further processes the operators, timeline, interaction, temporal patterns, and correlation analysis, and finally, the interface layer shows the user applications and interfaces.

The vectorization process in this approach is based on the latent Dirichlet allocation (LDA) to group some of the topics out of the data. LDA is a famous topic-discovery or topic-categorization approach that clearly separates the content into the clusters of similar data [57]. Each cluster contains similar information and the same direction in terms of meaning and content similarity or probability. Equation (1) presents the estimated topics *t* based on LDA and edge $q_{n}$, which transforms to the vector $\beta_{n}$ that provides the probability of $R(z_{m}|q_{n})$ for every topic.

(1) $$\beta_{n}=\left(R\left(z_{1}|q_{n}\right),R\left(z_{2}|q_{n}\right),\ldots ,R\left(z_{m}|q_{n}\right),\ldots ,R\left(z_{t}|q_{n}\right)\right)$$

For deciding the topic *t*, the perplexity model of LDA was applied. This model shows the model’s performance and how well it works. Equation (2) shows the process of the perplexity evaluation, where R(w) is the words’ probability output from the LDA model, and *i* is the number of words. Thus, the presented approach evaluates the LDA model perplexity for the vectorization.

(2) $$preplexity=q^{\frac{\Sigma log(R(w))}{i}}$$

Equation (3) presents the vertices $\beta_{m}$, which can be vectorized with a vector in *m* edges.

(3) $$A_{k}=\left(\beta_{1},\beta_{2},\ldots ,\beta_{m}\right)$$

Every vertex can have various numbers; in Equation (4), the vertex normalization is evaluated for the topic distribution.

(4) $$A_{k}=\frac{\left(\beta_{1},\beta_{2},\ldots ,\beta_{m}\right)}{I}$$

Based on the various generated vectors’ sizes, the last step is high dimensional. The presented system evaluates the feature relevancy composition to reduce the dimensions. The CFR algorithm is applied for the feature selection regarding the information that can discover topics’ degree of impact. Table 2 shows the details of the feature selection of the presented system.

### 3.2. Blockchain-Based Digital Forensic Analysis

**Figure 3:**
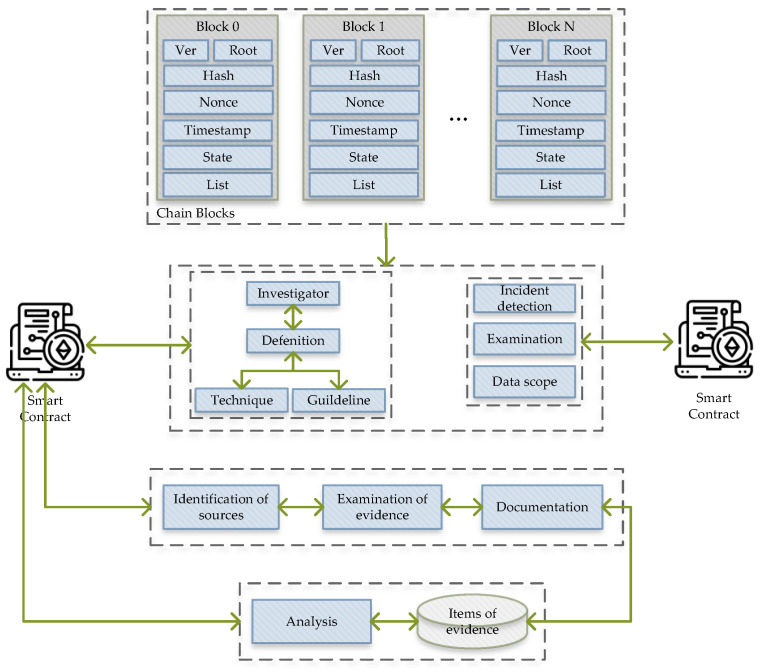
Blockchain-based evidence identification.

The blockchain approach for the digital forensics process is used to secure the forensic data in terms of transparency and performance. As shown in Figure 3, each entity links together, e.g., users, devices, evidence items, etc. The significant part to guarantee the digital evidence integrity is based on the hierarchy level in an investigation of chains.

There are three main processes defined for the DF investigation: applying a smart contract to perform the evidence analysis automatically, e.g., email analysis, signature or file analysis, etc, and providing better auditability by improving the investigation transparency, thereby reducing the costs and used resources and increasing connection stability between third parties.

## 4. Experimental Results and Development Environment

This section describes the details of the collected dataset, the system performance evaluation, the experiments, and the results of NLP and blockchain in forensic data analysis. Table 3 shows the details of the development environment for the digital forensic analysis.

### 4.1. Data Representation and Collection

The data collected in this system is from online social media (OSN), which implements the knowledge model by using ontologies and semantic web processes. The data collection is from famous social media websites, such as Facebook and Twitter, including comments, shares, news broadcasts, etc. The number of users in this environments is high and information sharing is very fast and impressive. Regarding this process, the records of fake shared information is also very high. In this process, we have used 80% of the collected dataset for the training set and 20% for the testing set. Table 4 presents the details of the collected dataset for this approach.

**Figure 4:**
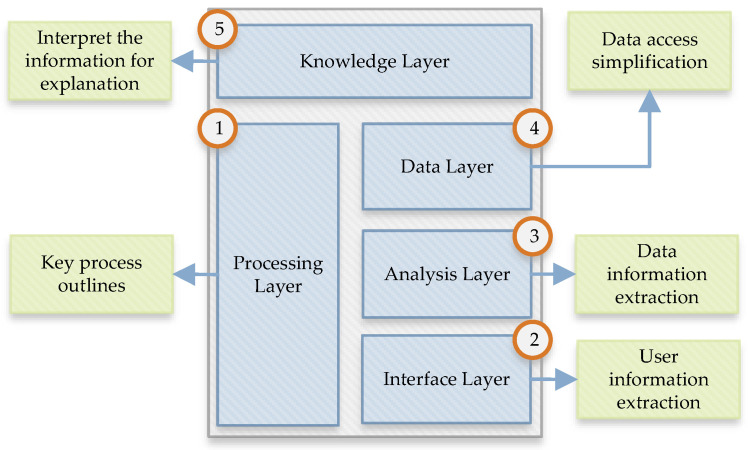
Multi-layered data processing.

The data layer is responsible for normalizing the provided data and storing them in persistent memory. This memory implements a design that is based on web schema. The unstructured data analysis requires a developed and customized tool for further processing. The analysis layer presents the analysis operators for the automatic process of social media contexts. The computerized analysis method is applied for quick data analysis and evaluation in this process. The decision-making process is a representative evaluation of the human examiner regarding various crimes in the social network evidence. Figure 4 shows the details of data classification and automation solutions.

Table 5 presents the details of the operators for the data analysis process. Eight operators use the subject and object correlation to analyze the dataset’s contents.

### 4.2. Performance Evaluation of the Proposed Online Digital Forensic Analysis

This part presents the performance evaluation of the proposed online forensic analysis. We have defined three metrics of precision *P*, recall *R*, and F-measure F1. In this process, we used the Random Forest algorithm to analyze this process and compare our results with the Decision Tree, Naive Bayes, Logistic Regression, and Support Vector Machine algorithms. The main reason for using Random Forest in this process is its good performance in terms of classification, as compared to the other algorithms [58]. Table 6 shows the details of each classifier’s performance for each fold. Equations (5)–(7) show the details of precision, recall, and f-measure in this process.

(5) $$Precision=\frac{T.Positive}{T.Positive+F.Positive}$$

(6) $$Recall=\frac{T.Positive}{T.Positive+F.Negative}$$

(7) $$Accuracy=\frac{T.Positive+T.Negative}{Total}$$

**Figure 5:**
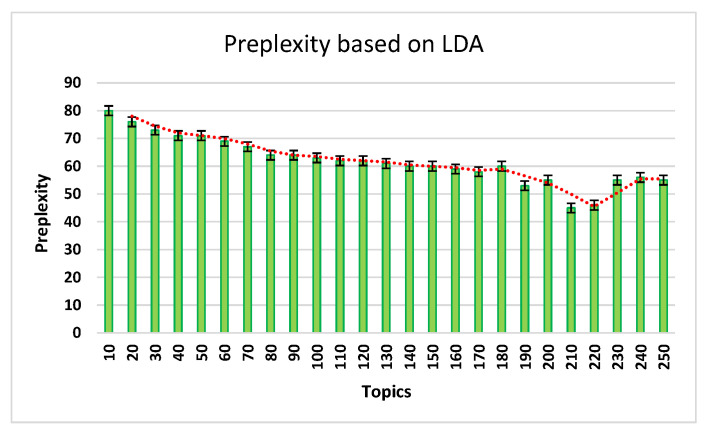
LDA-based preplexity records.

Figure 5 shows the perplexity records achieved from LDA and records 210 out of the 250 tested topics. Regarding this process, the data was vectorized for the 210 topics. In Figure 5, the x-axis presents the number of topics extracted from this process and the y-axis presents the perplexity of each topic category.

The next step is the cross-validation process. For each five-fold output, the classifier builds the *m* topics 1≤m≤210 in the training set and validates the highest performance record. Table 6 gives the details of defined three classifiers per fold, and Table 7 shows the further evaluations of the metrics’ average scores.

The presented system shows the benefits of feature selection in this process. Table 8 shows the details of the analysis with and without feature selection. The improvement of Random Forest’s performance is very visible. From the perspective of digital investigators, the feature selection is suitable to sort the related topics.

**Figure 6:**
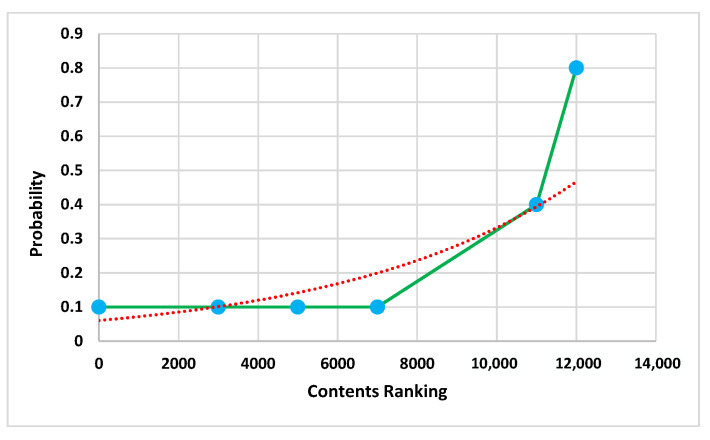
Topic probability analysis records.

The other benefit of applying sorted topics is identifying the communication between networks with a significant volume of data. Figure 6 shows the test set of 106 topics’ probability for each fold.

### 4.3. Security Analysis of Online Digital Forensic Based on Blockchain

Security in forensic data is one of the most important and challenging aspects in this area. We have used the blockchain framework for online digital forensic security analysis to improve this system’s transparency and rate of the trust according to the following steps:

- The first step is digital evidence identification. The aim of this is to identify the digital fingerprint of evidence. Furthermore, one fingerprint is generated to examine the event for every certain claim;
- Based on the timestamp and additional information, the fingerprint records are written into the evidence block and appended to the blockchain;
- In the blockchain network, every participant holds a copy of the evidence blockchain.

**Figure 7:**
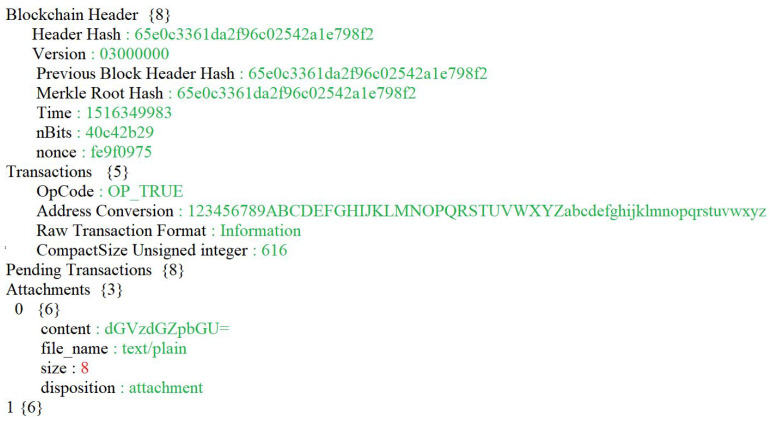
Evidence block in JSON script.

Figure 7 shows the JSON script for the evidence block.

**Figure 8:**
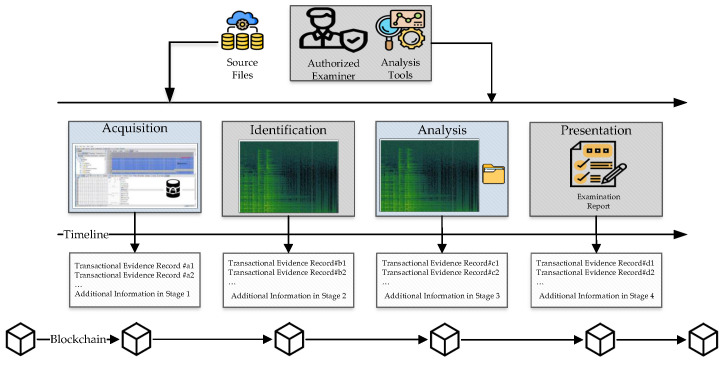
Details of the process of the blockchain framework for forensic analysis.

Figure 8 presents the details of using blockchain in forensic analysis. There are four main sections, namely, data acquisition, identification, analysis, and presentation. Regarding the timeline, the transactional evidence record is in the blockchain framework. In data acquisition section, all the related information is saved in the blockchain. In the identification section, suspicious files are saved in the blockchain too. In the analysis stage, by using the hash function, various file types are analyzed and stored in blockchain. The presentation stage writes all the reports and findings to the blockchain.

## 5. Conclusions

Social media communication is an important source of evidence for criminal investigations, such as fake news or fake election investigations. In this paper, we proposed the integration of NLP techniques with blockchain to improve the security and performance of online digital forensics. In terms of NLP, the LDA topic modeling, feature extraction, and data analysis were applied for a detail analysis of the collected dataset. The collected information is from multi-source social media platforms, which provides more opportunity for results comparisons in various aspects, as compared with other state-of-the-art approaches. The Random Forest algorithm was applied on a real-world dataset and compared with the other four algorithms, namely, the Decision Tree, Naive Bayes, Logistic Regression, and Support Vector Machine algorithms. The main reason for selecting Random Forest for this system is the higher performance of this algorithm in classification tasks and related processes. The concept of blockchain in this system is to improve system security and trace process changes. The defined system is processed in the Hyperledger Fabric framework. The blockchain framework gives the opportunity to the system process of saving and securing the results, as well as all the digital forensic processess and data, with details. Future studies in this topic can apply the presented system to cybercriminal activities and fraud to overcome the recent issues in this field. 

## Figures and Tables

**Table 1:** Comparison of the recent state-of-the-art DF methods.

Author	Proposed Approach	Advantages	Limitations
Choi et al. (2019) [52]	Digital forensic analysis for the Kakao Talk encrypted data.	Data recovery without requiring a password from a user.	Difficult to protect user-sensitive information.
Zhang et al. (2018) [53]	Digital forensic analysis for smart phone instant messaging.	Investigating the history of user messages in four Android mobile applications.	Limitation in communication mode for one-to- one contacts.
Du et al. (2020) [54]	Future of artificial intelligence in investigation of digital forensics.	Survey of automated evidence-processing methods based on AI techniques.	Image data with low quality is difficult to train and process further.
Xiao et al. (2019) [55]	Analysis of video- based evidence investigation of digital forensics.	Identification of forensics and link- establishment to investigate the objects.	Difficult to identify human face recognition, motion detection, etc.
Jadir et al. (2018) [56]	Digital forensic enhancement for document clustering,	Enhancing the document clustering performance for partitioning the criminal reports and text dataset.	Challenges of processing if the data numbers increase.

**Table 2:** Vertices before and after feature selection.

Before Feature Selection						
Samples	topic 1	topic 2	topic 3	topic 4	topic 5	y (label)
Vector 1	1	0	0	0	0	0
Vector 2	0	0	0.7	0.2	0.4	1
Vector 3	0.5	0.3	0.2	0	0.4	0
Vector 4	0	0.6	0	0	0.6	1
After Feature Selection						
Vectors	topic 1	topic 2	topic 3	y (label)		
Vector 1	1	0	0	0		
Vector 2	0	0	0.2	1		
Vector 3	0.5	0.3	0	0		
Vector 4	0	0.6	0	1		

**Table 3:** Development environment.

Module	Component	Description
Machine Learning	Operating System	Microsoft Windows 10
	CPU	Intel (R) Core (TM) i7-8700@3.20 GHz
	Main Memory	16GB RAM
	Core Programming Language	Python
	IDE	PyCharm Professional 2020
	ML Algorithm	Random Forest
Blockchain Framework	Operating System	Ubuntu Linux 18.04 LTS
	Docker Engine	Version 18.06.1-ce
	Docker Composer	Version 1.13.0
	IDE	Composer Playground
	Programming Language	Node.js

**Table 4:** Data information.

Data Type	Total Records
Facebook	5000
Twitter	6500
Blogs	6600
News	5500
Training Set	80%
Testing Set	20%

**Table 5:** Analysis operators list of the following processes.

Name of Operators	Details
Tweet Cloud	Object correlation method to provide the fast overview of users’ tweet topics.
Hashtag Cloud	Object correlation based on hashtags of user tweets.
Interaction Graph	Subject and object correlation for sorting contacts between the social graph of users with the highest communication frequency.
Interaction Frequency Analysis	Subject and objective correlation to perform the frequency analysis between two users and identify the relationship of the users’ communication.
Views Similarity	Rule-based correlation for nearest user-opinion identification.
Trace Operator	Linking the evidence to the entity.
Temporal Activity Graph	Using temporal correlation to analyze the user activity patterns in a defined period.
Geo-location Activity Graph	Object correlation for sorting the location based on the tagged online content.

**Table 6:** Different classifiers’ performance evaluation for each fold.

Fold	Metrics	Decision Tree	Naive Bayes	Logistic Regression	Random Forest	Support Vector Machine
1	P	0.6595	0.8254	0.9486	0.9846	0.6487
	R	0.7511	0.9700	0.7111	0.6911	0.7348
	F1	0.6667	0.6174	0.8611	0.7811	0.7794
2	P	0.7198	0.3541	0.6736	0.8947	0.4955
	R	0.5511	0.7511	0.4711	0.5948	0.6564
	F1	0.6944	0.5656	0.5611	0.7182	0.5836
3	P	0.6111	0.6993	0.8793	0.9831	0.6939
	R	0.6311	0.9300	0.7511	0.8334	0.8479
	F1	0.6825	0.6111	0.7622	0.7939	0.7749
4	P	0.5968	0.7986	0.8611	0.9444	0.6232
	R	0.8711	0.8711	0.7911	0.7746	0.7498
	F1	0.6929	0.6477	0.7994	0.8337	0.6949
5	P	0.6374	0.4058	0.7929	0.8478	0.5498
	R	0.5111	0.8711	0.7111	0.6964	0.7699
	F1	0.5990	0.6566	0.7633	0.7982	0.6479

**Table 7:** Average score of different classifiers.

Metrics	Decision Tree	Naive Bayes	Logistic Regression	Random Forest	Support Vector Machine
P	0.6449	0.5367	0.6393	0.0.9443	0.6279
R	0.6631	0.9191	0.6871	0.6943	0.7432
F1	0.6673	0.6197	0.7334	0.7611	0.6745

**Table 8:** Records with and without feature selection.

#	Metrics	Decision Tree	Naive Bayes	Logistic Regression	Random Forest	Support Vector Machine
With feature selection	P	0.6449	0.6367	0.8513	0.9443	0.6279
	R	0.6631	0.9191	0.6871	0.6943	0.7432
	F1	0.6673	0.6197	0.7534	0.7611	0.6745
Without feature selection	P	0.6293	0.6176	0.8122	0.8321	0.4574
	R	0.6171	0.5351	0.6791	0.5467	0.6831
	F1	0.6372	0.5779	0.6974	0.6998	0.5445

## Data Availability

Not applicable.
